# Synergy of Tetracyclines and Potassium Azeloyl Diglycinate (Azeloglycine) in Hydrogels: Evaluation of Stability, Antimicrobial Activity, and Physicochemical Properties

**DOI:** 10.3390/ijms26115239

**Published:** 2025-05-29

**Authors:** Agnieszka Kostrzębska, Adam Junka, Witold Musiał

**Affiliations:** 1 Department of Physical Chemistry and Biophysics, Pharmaceutical Faculty, Wroclaw Medical University, Borowska 211, 50-556 Wroclaw, Poland; agnieszka.kostrzebska@umw.edu.pl; 2 Platform for Unique Models Application P.U.M.A., Department of Pharmaceutical Microbiology and Parasitology, Wroclaw Medical University, Borowska 211, 50-556 Wroclaw, Poland; adam.junka@umw.edu.pl

**Keywords:** acne, *S. aureus*, tetracyclines, potassium azeloyl diglycinate, AMPD, skin sebum, hydrogel, HPLC, FTIR, *Galleria mellonella* larvae

## Abstract

Acne vulgaris is one of the most common dermatological diseases and has a complex etiology. Despite the wide range of available therapeutic options, modern and effective solutions are still being sought, particularly in the area of topical therapy. The aim of this study was to develop hydrogel formulations that provide stability for the antibiotics they contain—tetracycline or chlortetracycline enriched with azeloglycine—the latter an ingredient supporting acne-prone skin care. The physicochemical parameters, stability, and antimicrobial activity of the obtained formulations were analyzed. HPLC analysis showed that tetracycline exhibited greater stability than chlortetracycline, especially in mildly acidic and neutral environments. The addition of azeloglycine improved the rheological properties of the hydrogels, reduced tetracycline degradation under alkaline conditions, and enhanced the penetration of active ingredients into the model sebum. All tested formulations demonstrated antimicrobial activity against *Staphylococcus aureus*. In the artificial sebum biofilm model, hydrogels containing azeloglycine more effectively reduced staphylococcal biofilm mass. No formulations showed toxicity towards *Galleria mellonella* larvae. The results indicate the potential usefulness of the developed hydrogels as modern multifunctional formulations for topical acne treatment. Hydrogel formulations containing tetracycline and azeloglycine may represent a promising future anti-acne preparation exhibiting synergistic antibacterial, anti-inflammatory, and sebum-cleansing effects.

## 1. Introduction

Acne vulgaris is one of the most common skin diseases and affects a significant proportion of the population, especially young people, with major impacts on their quality of life and psychological state. This disorder is characterized by a complex pathogenesis that includes disturbances in the balance of the skin microbiota [1,2,3]. In the course of this dermatosis, the pilosebaceous apparatus becomes colonized by microorganisms such as *Cutibacterium acnes*, *Staphylococcus aureus*, *Staphylococcus epidermidis*, and others, which in turn leads to overproduction of sebum, blockage of the sebaceous glands, and the development of a controlled condition that results in characteristic skin lesions [3,4,5,6]. The intensity of clinical symptoms is variable, and in about 60–80% of cases, acne is mild, and topical therapy brings satisfactory results. Severe forms of the disease require systemic treatment, which is associated with the risk of adverse effects [7,8,9,10]. This fact underscores the need to search for new therapeutic strategies characterized by both high efficacy and safety while at the same time meeting the needs of patients in terms of convenience of use. Despite progress in the development of dermatological drugs, acne vulgaris remains a clinical challenge. The need to develop innovative treatment methods, especially those that consider the role of the skin microbiome and the specificity of local inflammatory processes, is an area of particular interest in modern dermatology and pharmacy.

This study focused on tetracycline antibiotics, such as tetracycline hydrochloride and chlortetracycline hydrochloride, which are used for the oral treatment of acne vulgaris and for the topical treatment of bacterial infections, such as purulent skin problems [7,11,12,13,14]. In addition to the known antibacterial effects of tetracycline antibiotics, their anti-inflammatory activity is critical, making them particularly effective in reducing inflammatory acne lesions [15,16,17]. Potassium azeloyl diglycinate, known as azeloglycine, has been incorporated in formulations in order to develop dermatological products with a multidirectional mechanism of action. This compound, widely used in the care of acne-prone skin, has many biological effects, including anti-inflammatory and antibacterial properties and the ability to lighten acne scars [18,19,20,21,22]. Another important ingredient in formulations is the alcoholamine 2-amino-2-methyl-1,3-propandiol (AMPD), which is characterized by its ability to interact with the components of model sebum and can potentially be used in the process of cleansing the skin of sebum deposits [23,24].

In this study, a comprehensive analysis and comparison of hydrogel formulations containing tetracycline or chlortetracycline, both with and without the addition of azeloglycine, was performed. The evaluation included characterization of physicochemical properties and microbiological activity of the tested formulations. The main objective of the work was to verify the possibility of developing stable, innovative dermatological hydrogels with a multidirectional profile of action, including antibacterial, anti-inflammatory, and seboregulatory properties, with potential use in the local treatment of acne.

## 2. Results

### 2.1. pH Values of Hydrogel Formulations

In order to obtain hydrogels with different pH, the acrylic acid polymer was neutralized with different amounts of the weak base 2-amino-2-methyl-1,3-propanediol (AMPD). Hydrogels identified as group 1 (1TA, 1TB, 1ChA, 1ChB) had a slightly acidic pH of around 6.30. Hydrogels identified as group 2 (2TA, 2TB, 2ChA, 2ChB) had a higher pH of around 7.30, while hydrogels in group 3 (3TA, 3TB, 3ChA, 3ChB) had the highest, alkaline pH, with values close to 8.20. Table 1 shows the pH values of the preparations.

### 2.2. Stability Evaluation of Antibiotics Based on HPLC Chromatography

**Figure 1:**
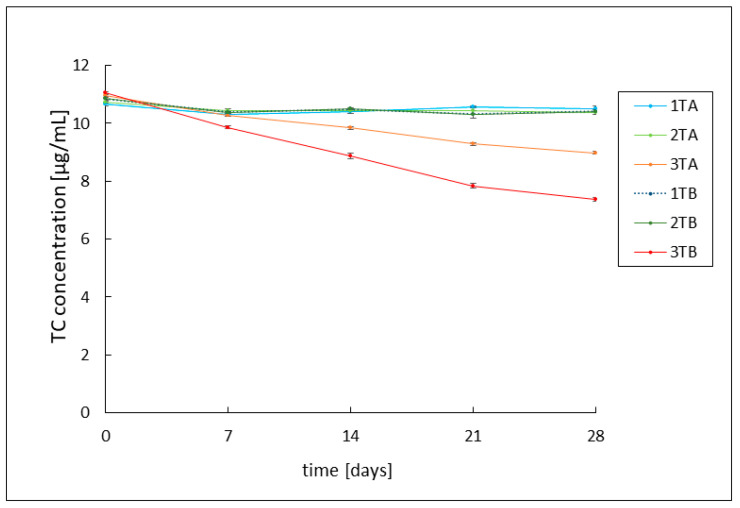
Tetracycline (TC) concentration in 1TA, 1TB, 2TA, 2TB, 3TA, and 3TB formulations over 28 days. The abbreviations conform to Table 1.

In this study, the stability of hydrogels containing tetracycline and chlortetracycline was evaluated over a period of 28 days by HPLC analysis. Measurements were made every 7 days. All formulations were stored under optimal stability conditions at 4 °C and protected from light, which is consistent with our previous studies on tetracycline [25]. The tetracycline hydrochloride preparations 1TA, 1TB, 2TA, and 2TB showed high stability of the antibiotic throughout the analysis period, with drug concentration values oscillating around an initial value of approximately 10.5 μg/mL. The presence or absence of azeloglycine did not affect the stability of tetracycline. In the case of the basic hydrogels 3TA and 3TB, a progressive degradation of the antibiotic was observed. The azeloglycine-containing hydrogel 3TA showed greater stability, with the drug concentration value decreasing from about 10.9 μg/mL on the first day of observation to about 8.9 μg/mL on day 28. The 3TB hydrogel showed less stability, with a final antibiotic concentration of approximately 7.4 μg/mL on day 28. The results obtained are shown in Figure 1.

**Figure 2:**
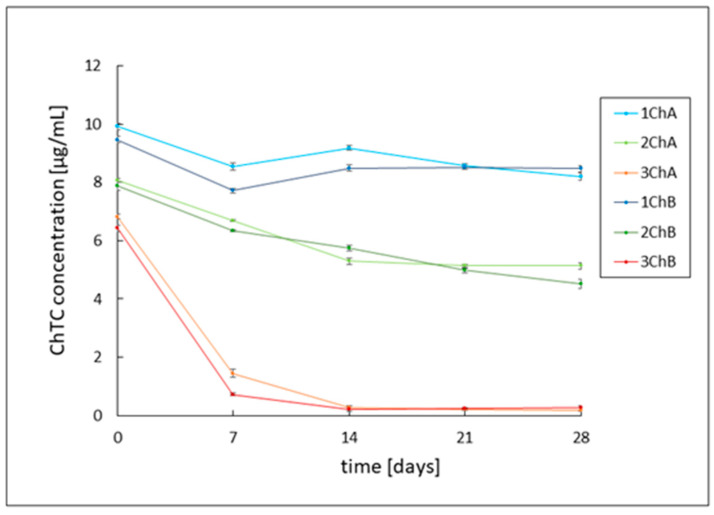
Chlortetracycline (ChTC) concentration in 1ChA, 1ChB, 2ChA, 2ChB, 3ChA, and 3ChB formulations over 28 days. The abbreviations conform to Table 1.

The stability of chlortetracycline in the developed formulations showed varied results. Despite consistent hydrogel and sample preparation methods for HPLC analysis, only the slightly acidic 1ChA and 1ChB gels achieved the expected initial drug concentration of ca. 10 μg/mL. For the 2ChA and 2ChB preparations the initial concentration of chlortetracycline was around 8 μg/mL, while for the alkaline gels 3ChA and 3ChB, it was around 6.6 μg/mL. Chlortetracycline has been shown to be more unstable than tetracycline. The decrease in the initial concentration values in the samples may be related to the breakdown of the antibiotic or its reversible epimerization. The slightly acidic gels 1ChA and 1ChB showed relatively high stability, with a slight decrease in chlortetracycline concentration to about 8.4 μg/mL on the last day of observation. Gels with pH close to neutral—2ChA and 2ChB—showed lower stability, with a slow decrease in ChTC concentration from about 8 μg/mL to just below 5 μg/mL. The greatest degradation was observed in alkaline gels 3ChA and 3ChB, where the drug concentrations decreased to 1.45 μg/mL for gel 3ChA and 0.73 μg/mL for gel 3ChB in the first week of observation and reached values close to 0 μg/mL in the following weeks. The course of changes in antibiotic concentrations in these preparations was not affected by the presence of azeloglycine. The results of this analysis are shown in Figure 2.

### 2.3. Assessment of Rheological Parameters

The shear-thinning properties of non-Newtonian fluids were exhibited by the formulations studied, which were based on an acrylic acid polymer [26,27,28,29,30]. All measurements were carried out under the same conditions, at 37 °C, with gradually increasing shear rates ranging from 0.38 s^−1^ to 3.84 s^−1^. The viscosity curves for all formulations, regardless of the type of antibiotic used, followed a similar pattern. Azeloglycine was found to have a considerable effect on the viscosity of the formulations tested, with its addition significantly reducing the viscosity of the formulations.

All formulations without azeloglycine showed similar viscosity value regardless of pH. At an initial shear rate of 0.38 s^−1^, the viscosity oscillated around 213,000 mPas for samples 1TB and 2ChB and 221,000 mPas for samples 2TB and 1ChB. The 3TB and 3ChB formulations had a slightly lower viscosity of around 205,000 mPas. As the shear rate increased, the viscosity decreased, eventually reaching around 46,000 mPas for the 1TB and 2ChB gels, around 47,000 mPas for 2TB, 48,000 mPas for 1ChB, 44,000 mPas for 3TB, and just below 42,000 mPas for 3ChB.

**Figure 3:**
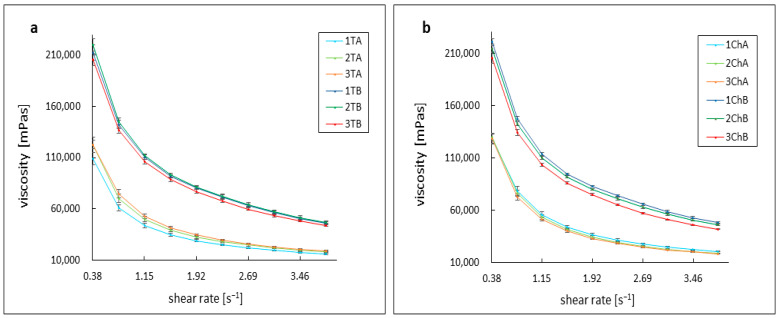
Dynamic viscosity curves at 37 °C for formulations of tetracycline (**a**) and chlortetracycline (**b**). The abbreviations conform to Table 1.

Mutual comparable viscosity values were also observed for gels containing azeloglycine, regardless of pH. However, these values were significantly lower compared to formulations without azeloglycine. At a shear rate of 0.38 s^−1^, the viscosity was approximately 128,000 mPas for 1ChA, 2ChA, and 3ChA gels, approximately 122,500 mPas for 2TA and 3TA, and 108,800 mPas for 1TA. With increasing shear rate, the viscosity decreased to final values of about 20,400 mPas for 1ChA, about 18,600 mPas for 2ChA and 3TA, about 18,000 mPas for 2TA and 3ChA, and 15,800 mPas for 1TA. The course of the viscosity curves is shown in Figure 3.

### 2.4. Evaluation of the Activity of Preparations Against Model Skin Sebum

**Figure 4:**
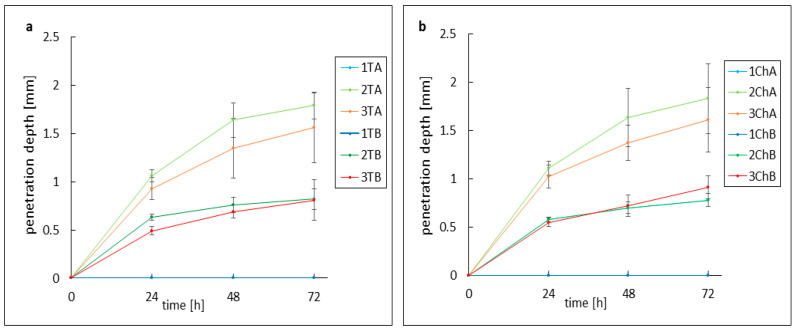
Changes in layer depth of the resulting products during 72 h of observation for preparations containing tetracycline (**a**) and chlortetracycline (**b**). The abbreviations conform to Table 1.

**Figure 5:**
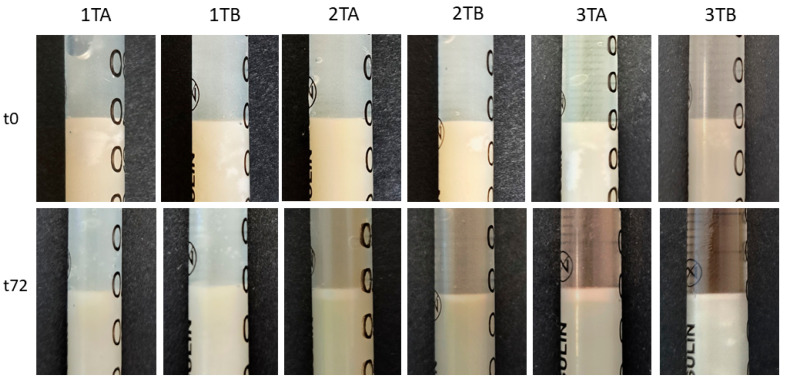
Presentation of changes in the sebum surface under the influence of tetracycline-containing hydrogels (TA and TB) at 0 h and 72 h after the start of the observations. The abbreviations conform to Table 1.

**Figure 6:**
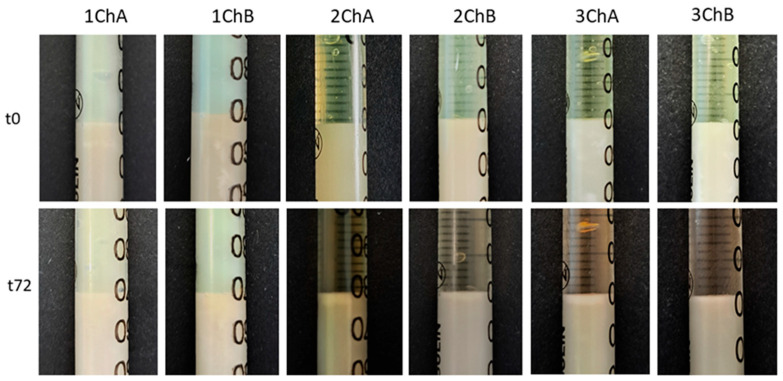
Presentation of changes in the sebum surface under the influence of chlortetracycline-containing hydrogels (ChA and ChB) at 0 h and 72 h after the start of the observations. The abbreviations conform to Table 1.

The response of AMPD alcoholamine to model skin sebum components was observed over 72 h, with changes recorded every 24 h. As shown in previous studies, the activity of hydrogel alcoholamine towards model sebum components is dependent on the pH of the formulation [31]. The type of antibiotic used had no effect on the rate of reaction of the hydrogel with the sebum components—pH was the determining factor. For the slightly acidic gels 1TA, 1TB, 1ChA, and 1ChB, no reaction was observed throughout the study period. In the other formulations, a saponification reaction was observed, manifested by the formation of a white layer of products at the sebum–hydrogel interface. For all hydrogels, the highest reaction rate was recorded in the first 24 h, which may indicate a depletion of free alcoholamine in the hydrogels over time. A second factor influencing the efficiency of the AMPD reaction with sebum was the presence of the additional ingredient azeloglycine. Significant differences were found between gels with and without azeloglycine: the presence of azeloglycine increased the reactivity of the formulation with sebum. The activity of neutral and slightly alkaline pH hydrogels without azeloglycine, i.e., 2B, 3B, 2ChB, and 3ChB, was similar. After 24 h, the depth of the product layer was approximately 0.55 mm for the 2TB, 2ChB, and 3ChB gels and 0.49 mm for the 3TB gel. After 72 h, the depth of the product layer increased to almost 0.8 mm for these gels. Deeper product layers were observed for 2TA, 3TA, 2ChA, and 3ChA gels. Both 2TA and 2ChA formulations showed slightly higher activity than the more alkaline 3TA and 3ChB gels. After 24 h, the depths of the product layer were: 2TA 1.06 mm, 3TA 0.93 mm, 2ChA 1.11 mm and 3ChA 1.02 mm. After 48 h, the depth of the product layer for the 2TA and 2ChA gels was approximately 1.64 mm, and for the 3TA and 3ChA gels was approximately 1.36 mm. After 72 h, the depth was about 1.80 mm for 2TA and 2ChA gels and about 1.6 mm for 3TA and 3ChA gels. Changes in the depth of the resulting product layer are shown in Figure 4. Figure 5 and Figure 6 compare the models analyzed: when the hydrogels were applied to the sebum layer and after 72 h, showing the resulting layer of reaction products.

### 2.5. Attenuated Total Reflectance–Fourier Transform Infrared Spectroscopy Analysis

In this study, detailed Fourier transform infrared (FTIR) analysis was carried out to characterize the ingredients contained in the hydrogels and the prepared hydrogel formulations. The study included spectroscopic analysis of the components, i.e., Carbopol polymer, AMPD alcoholamine, antibiotics, and azeloglycine, for detailed characterization. The FTIR spectra of the prepared hydrogels in different formulations were then measured. This allowed the determination of changes in absorption spectra resulting from interactions between hydrogel components, such as neutralization of Carbopol by AMPD and spatial lattice formation.

**Figure 7:**
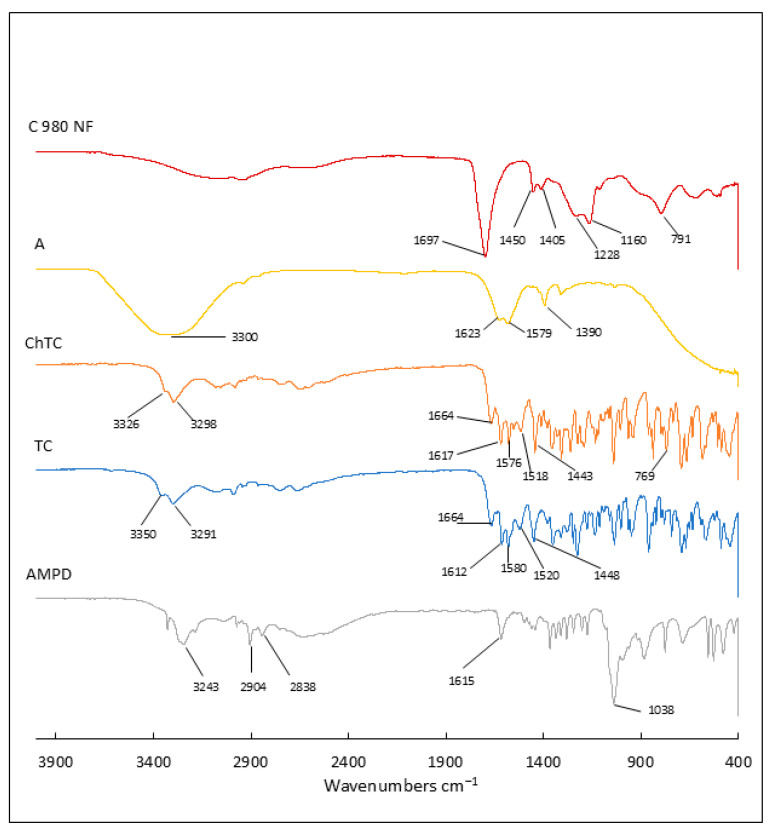
Fourier transform infrared spectroscopy with attenuated total reflectance (ATR–FTIR): spectra of individual components: Carbopol 980 NF (C 980 NF), azeloglycine (A), chlortetracycline hydrochloride (ChTC), tetracycline hydrochloride (TC) and 2-amino-2-methyl-1,3-propandiol (AMPD).

Figure 7 shows the ATR–FTIR spectra of the commercial components. In the FTIR spectrum of Carbopol 980 NF, an acrylic acid polymer, the peaks present confirmed the presence of functional groups characteristic of this polymer. The intense band at 1697 cm^−1^ may be related to symmetric C=O stretching vibrations of carbonyl groups, confirming the presence of carboxyl groups. Lower-intensity bands at 1450 cm^−1^ and 1405 cm^−1^ may correspond to scissor vibrations of C–H bonds in CH_2_ methyl groups in the aliphatic chain of the polymer [32,33,34]. The peak at 1405 cm^−1^ may also indicate O–H bending vibrations in carboxyl groups [35]. At 1228 cm^−1^, there is an intense band that may be related to the stretching vibrations of the C–O bonds of the acrylates, while at 1160 cm^−1^ there is an intense band that may be related to the C–O–C stretching vibrations that may be present in the structure of the cross-linked polymer [32,35]. The band at 791 cm^−1^ may indicate bending vibrations of C=CH bonds, which in the case of Carbopol may indicate residual double bonds in the polymer structure [35,36].

In the case of the AMPD alcoholamine spectrum at 3243 cm^−1^, the intense band may indicate O–H stretching in the hydroxyl groups or N–H stretching in the amine groups [37,38]. The band at 2904 cm^−1^ may refer to C–H stretching vibrations in aliphatic groups, while the band at 2838 cm^−1^ may refer to asymmetric C–H stretching vibrations, but mainly related to the methyl group. At 1615 cm^−1^, the rather intense band may be related to the vibration of the N–H scissors in the amine groups –NH_2_ [37]. The intense band at 1038 cm^−1^ may indicate the presence of C–N stretching vibrations characteristic of aliphatic amines [38].

The spectra of tetracycline and chlortetracycline show many similarities. Chlortetracycline retains most of the characteristics of the tetracycline spectrum; however, the chlorine atom present on the 7th C atom of the D ring causes some changes in the FTIR spectrum. Bands at 3350 cm^−1^, 3291 cm^−1^ for TC and 3326 cm^−1^, 3298 cm^−1^ for ChTC are present, which may be related to the stretching vibrations of the –OH and –CONH bonds of the hydroxyl and amide groups of ChTC [39,40,41]. Bands at 1664 cm^−1^ (TC, ChTC) indicate C=O stretching and NH_2_ deformation vibrations of the amide group I [40,41,42]. The bands at 1612 cm^−1^ (TC) and 1617 cm^−1^ (ChTC) and 1580 cm^−1^ (TC) and 1576 cm^−1^ (ChTC) can be attributed to C=O stretching vibrations in the A and C rings, respectively [40,42,43,44]. Bands at 1520 cm^−1^ (TC) and 1518 cm^−1^ (ChTC) may indicate stretching vibrations of C–NH_2_ amide II [40,44,45]. The intense band at 1448 cm^−1^ (TC) and 1443 cm^−1^ (ChTC) may indicate C–C stretching vibrations [42,43,44,45]. The presence of a chlorine atom in the chlortetracycline molecule results in the presence of a band at 769 cm^−1^ that is unique to this antibiotic and is characteristic of C–Cl stretching vibrations [46].

The spectrum of azeloglycine is characterized by a broad band in the range 3366–3292 cm^−1^, which can be attributed to stretching vibrations of the O–H groups from the presence of water (associated with the solution form) or from azelaic acid, as well as N–H vibrations from the amino groups of glycine. The band at 1623 cm^−1^ may correspond to C=O stretching vibrations of the carbonyl groups of azelaic acid or N–H deformation vibrations of the –NH_2_ amino groups of glycine. The band at 1579 cm^−1^ may originate from N–H deformation vibrations in the amino groups or asymmetric COO^−^ vibrations. The band at 1390 cm^−1^ may correspond to symmetric COO^−^ vibrations or deformation vibrations of methyl groups of –CH_2_ [46,47,48].

**Figure 8:**
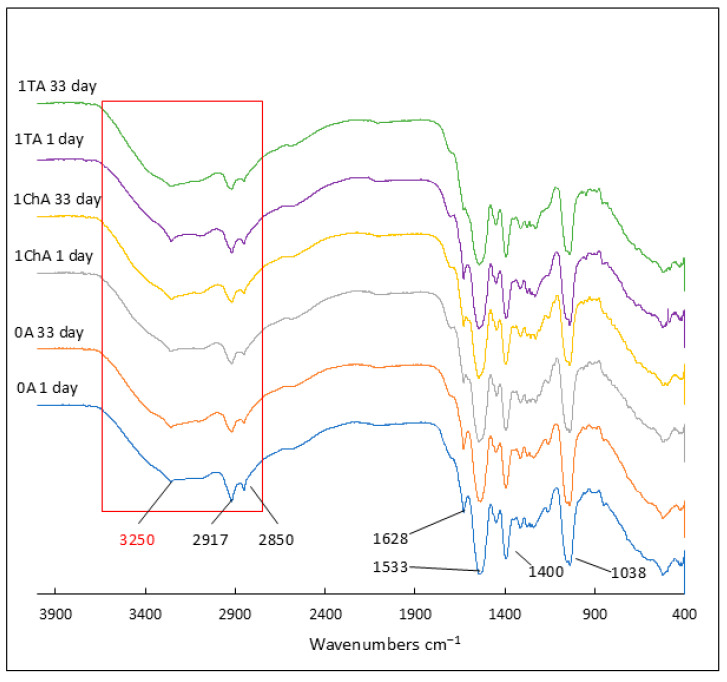
Attenuated total reflectance–infrared spectroscopy (ATR–FTIR): spectra of formulations containing azeloglycine 1 day and 33 days after lyophilization. Formulation 0A is devoid of antibiotic, 1TA contains tetracycline, and 1ChA contains chlortetracycline.

**Figure 9:**
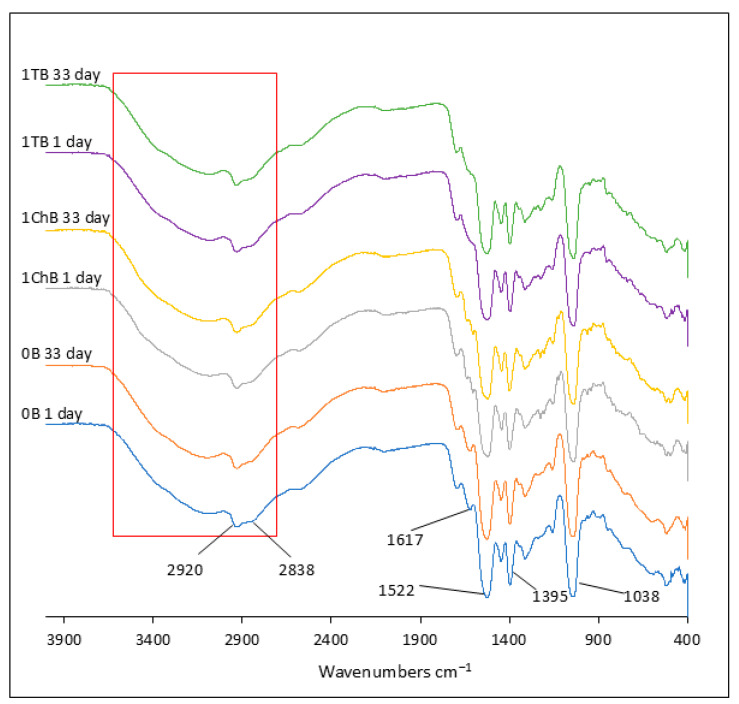
Attenuated total reflectance–infrared spectroscopy (ATR–FTIR): spectra of formulations without azeloglycine 1 day and 33 days after lyophilization. Formulation 0B is devoid of antibiotic, 1TB contains tetracycline, and 1ChB contains chlortetracycline.

In the case of hydrogels, all formulations that had previously undergone lyophilization were analyzed in detail by FTIR. The samples were analyzed twice: on the first day and 33 days after lyophilization. The spectra obtained are shown in Figure 8 and Figure 9. Due to the high similarity of the spectra in all the preparations studied, the figures show representative spectra for the preparations in the group labeled 1. In all hydrogel spectra, it was observed that the bands characteristic of the active ingredients, such as tetracycline, chlortetracycline, and azeloglycine, were obscured by the hydrogel-specific bands.

Upon hydration and neutralization of Carbopol, the FTIR spectrum undergoes some changes. Hydration and neutralization affect the conformation of the polymer, which can lead to subtle changes in the FTIR spectrum, such as changes in the intensity and position of bands associated with carbon skeletons and functional groups. A broad band appears at 3200–3600 cm^−1^, indicating the presence of water in the gel, associated with O–H stretching vibrations [49]. It is very characteristic that this band in all A hydrogels containing azeloglycine is less intense than the band in the spectra of B preparations lacking this component. These differences are highlighted in Figure 8 and Figure 9 with a red box. In all azeloglycine-containing preparations, this band is smaller and a small peak at about 3250 cm^−1^ is visible, which may be due to N–H stretching vibrations in the amine groups derived from AMPD [37,38]. In azeloglycine-free gels, this peak is covered by a more intense band in the 3200–3600 cm^−1^ range. The less intense band, originating from water, may indicate a possible effect of azeloglycine in reducing the water content of the polymer network. The bands at about 2917 cm^−1^ and about 2850 cm^−1^ may be related to stretching vibrations of C–H groups in methyl (–CH_3_) or methylene (–CH_2_) chains [49]. The presence of these bands may be related to AMPD alcoholamine or organic residues in the polymer structure. These bands were more noticeable in group A hydrogels containing azeloglycine. Upon neutralization, the carboxyl groups transform into ionized forms of COO^−^. This resulted in a shift in bands from 1697 cm^−1^ to lower values for all analyzed hydrogels, visible as a small band at about 1628 cm^−1^, an intense band at about 1550–1520 cm^−1^, and the appearance of a new band near 1400 cm^−1^, corresponding to symmetric COO^−^ stretching vibrations. The band seen at 1038 cm^−1^ may indicate the presence of C–N stretching vibrations, confirming the presence of AMPD in the polymer network [38,49]. There were no differences in the FTIR spectra of the hydrogels analyzed the day after freeze–drying or after 33 days, which may indicate the high stability of the polymer network and the lack of significant changes in its structure over time.

### 2.6. Microbiological Activity of the Formulations

**Figure 10:**
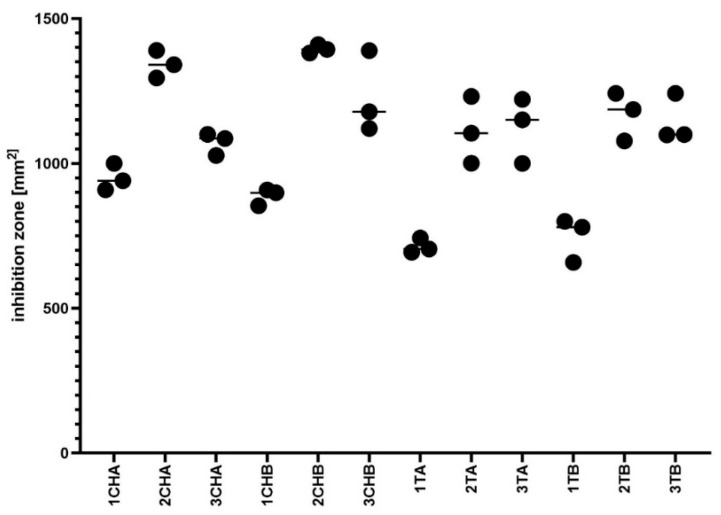
Assessment of staphylococcal growth inhibition zone after exposure to hydrogels using modified disk diffusion method.

**Figure 11:**
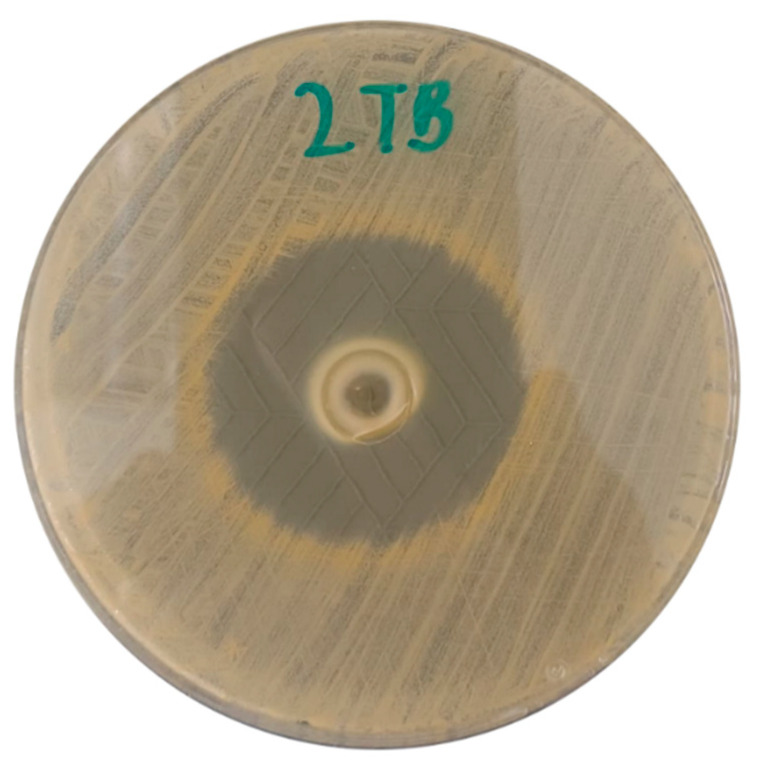
Exemplary visualization of staphylococcal growth inhibition on 90 mm petri dish after exposure to one of the 2TB hydrogel formulations.

In the first step of biological assessments, we evaluated the antibacterial activity of the hydrogels using a modified disk diffusion method against *S. aureus* ATCC 6538. As seen in Figure 10 and Figure 11, all tested hydrogels exhibited zones of growth inhibition. Notably, the inclusion of azeloglycine did not result in statistically significant differences in antimicrobial activity compared to formulations without this compound, indicating that azeloglycine may not enhance the antibacterial properties in this model.

**Figure 12:**
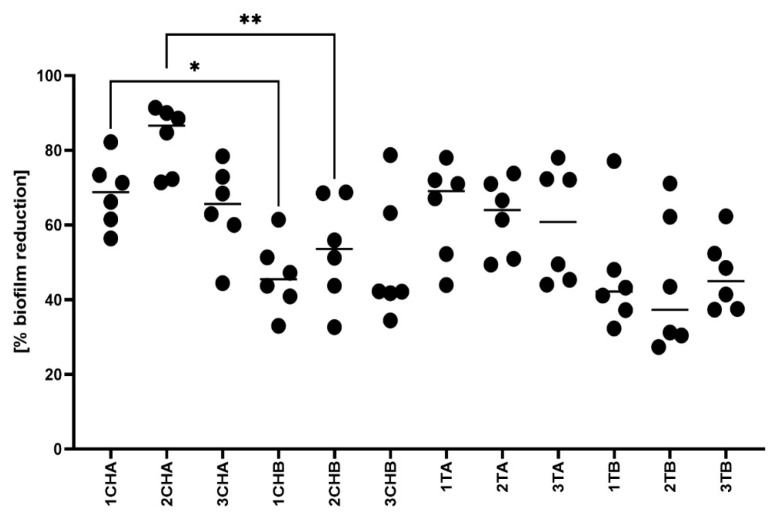
Assessment of staphylococcal biofilm reduction in the artificial sebum.

**Figure 13:**
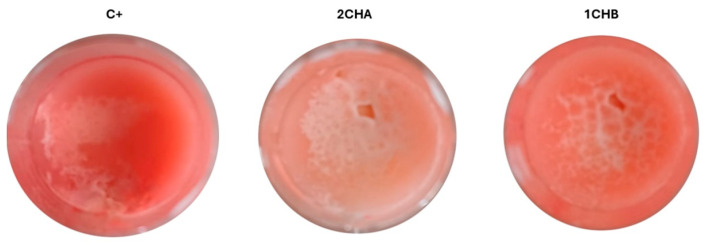
Visualization of biofilm reduction in the model using artificial sebum.

Next, we assessed the anti-biofilm efficacy of the hydrogels in a model simulating artificial sebum, which mimics the skin environment. The results, presented in Figure 12 and visualized in Figure 13, revealed statistically significant differences between formulations 1ChA and 1ChB, as well as between 2ChA and 2ChB. These findings suggest that specific compositional changes in the hydrogels impact their ability to reduce established *S. aureus* biofilms.

**Figure 14:**
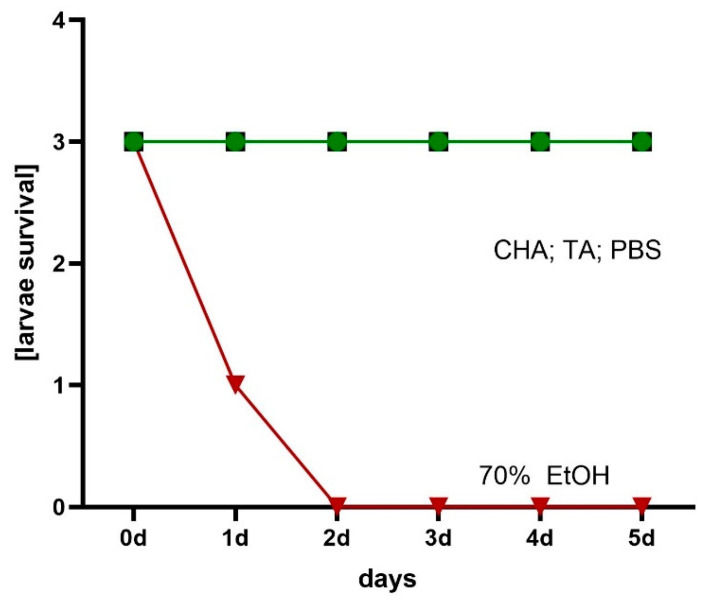
Assessment of hydrogels’ local cytotoxicity towards *G. mellonella* larvae.

Finally, the local cytotoxicity of the hydrogels was evaluated using the *Galleria mellonella* larva model. As shown in Figure 14, the ChA and TA hydrogels did not exhibit any toxic effects over the 5-day observation period. The larvae remained viable, with no signs of melanization or loss of motility, indicating a favorable safety profile for these formulations in vivo.

## 3. Discussion

Acne vulgaris is a common dermatosis among adolescents, with a prevalence of more than 80%. The vast majority of patients experience mild lesions that can be effectively treated with ointments, creams, hydrogels, and other topical preparations [1,50,51]. Topical therapy is safer and avoids many of the side effects associated with oral medications [7]. Topical acne treatments include antibiotics and retinoids, as well as keratolytic, comedolytic, discoloration, and scar-lightening agents such as benzoyl peroxide, salicylic acid, and azelaic acid and its derivative, potassium azeloyl diglycinate (azeloglycine) [8,9,10]. Hydrogel formulations are increasingly being chosen for topical therapy in the treatment of skin diseases, including acne, due to their unique properties, which are superior to traditional ointments. They are characterized by better penetration of active ingredients deep into the skin, which increases the effectiveness of therapy. In addition, they provide adequate moisture without leaving an oily film, which is important for patients with acne-prone skin. Their light texture promotes better absorption and application comfort. Hydrogels also minimize the risk of irritation and allergic reactions [52,53,54].

Two antibiotics from the tetracycline group—tetracycline and chlortetracycline—which are not standard preparations used in the topical treatment of acne, were selected for the present study of gels with potential anti-acne activity. Tetracyclines are a group of antibiotics with a broad spectrum of antibacterial and anti-inflammatory activity, making them valuable therapeutic agents in dermatology. Their therapeutic versatility is due to their ability to treat both bacterial infections and inflammatory dermatological diseases. They are mainly used in oral therapy. The mechanism of antimicrobial action of tetracyclines is based on binding to the 30S subunit of the bacterial ribosome, which leads to inhibition of protein translation and prevents synthesis of proteins necessary for bacterial growth. As a result, tetracyclines effectively inhibit bacterial growth and proliferation [11,12,55]. In addition to their antibacterial activity, tetracyclines also exhibit anti-inflammatory activity independently of their bacteriostatic activity. The anti-inflammatory mechanism involves inhibiting the activity of matrix metalloproteinases (MMPs) and reducing the production of proinflammatory cytokines, which helps to reduce tissue inflammation [7,15,16,17,56]. Tetracycline and its derivatives are commonly used to treat acne by the oral route, while chlortetracycline is mainly used in the topical treatment of purulent skin infections and in ophthalmology, where it is used in an eye ointment [7,11,12,13,14,57,58,59,60,61,62]. Both antibiotics are used to treat skin infections with the *S. aureus* strain [63]. The beneficial properties of these antibiotics, which include broad-spectrum antimicrobial activity and efficacy in reducing inflammatory processes, make tetracycline and chlortetracycline promising active ingredients in the development of new anti-acne formulations.

The microbiological activity of tetracycline and chlortetracycline has been combined with the highly beneficial dermatological properties of azeloglycine. It is a derivative of azelaic acid that exhibits all its beneficial therapeutic and skin care properties, such as antibacterial and anti-inflammatory, and inhibition of skin keratosis and lightening of acne hyperpigmentation [18,19,20,21,22]. Thanks to the structural modification with the introduction of two glycine molecules, it has much better physicochemical properties than azelaic acid: it dissolves much better in water, has no irritating effects, and shows compatibility with other ingredients [21,64,65]. In our previous studies evaluating the effects of azelaic acid and its derivatives on the physicochemical properties of hydrogels and the stability of tetracycline, azeloglycine was shown to be more effective. The presence of azelaic acid resulted in increased degradation of tetracycline and a significant decrease in the viscosity of the hydrogel, which may adversely affect the application process of the product on the skin. On the other hand, azeloglycine provided more favorable stability of the antibiotic and better rheological properties, indicating its potential as an ingredient to improve the performance and durability of anti-acne formulations [66].

A critical aspect of the formulations developed was to ensure the stability of the antibiotics they contained. Several studies of other authors detailed the degradation of tetracyclines. Tetracycline antibiotics are highly sensitive compounds that are susceptible to degradation when exposed to extreme pH, light, and high temperatures [67,68]. The nature of the degradation processes, including epimerization or dehydration, is similar for both tetracycline and chlortetracycline [69,70,71,72]. In a weakly acidic environment, tetracycline antibiotics undergo reversible epimerization to 4-epitetracycline and 4-epichlortetracycline without microbial activity [73,74,75]. In a strongly acidic environment at pH values below 2, the parent compound dehydrates to form anhydrotetracycline or anhydrochlortetracycline and then epimerizes in the case of tetracycline to 4-epianhydrotetracycline, which is already a nephrotoxic compound causing reversible Fanconi syndrome [74,76]. Differences in the degradation processes of tetracycline and chlortetracycline become apparent in alkaline environments. At pH values above 8, tetracycline is converted to quinone compounds [77,78,79]. In the case of chlortetracycline, isochlortetracycline is formed, which then undergoes further degradation, leading to the formation of additional breakdown products [71,72,80]. Both antibiotics also undergo degradation processes when exposed to light. The photodegradation of TC and ChTC molecules is similar, but faster in an alkaline environment, resulting in the formation of phototoxic products such as lumitetracycline [72,79,81,82,83,84].

In the present study, a 28-day stability analysis of both antibiotics was performed based on HPLC analysis. There was a noticeable difference in the stability of individual antibiotics. Tetracycline showed significantly higher stability in all formulations over the 28-day period. The concentration values of the drug in weakly acidic and neutral pH formulations, namely 1TA, 1TB, 2TA and 2TB, oscillated around an initial value close to 10.5 μg/mL. No effect of azeloglycine on antibiotic stability was observed. In the case of alkaline hydrogels (3TA and 3TB), progressive degradation of tetracycline was observed, with significantly higher stability of azeloglycine-containing formulations. The concentration of tetracycline in the 3TA gel decreased from an initial value of 10.9 μg/mL to 8.9 μg/mL on the 28th day of observation, while in the 3TB gel it decreased to 7.4 μg/mL on the last day. This may indicate a beneficial effect of azeloglycine in reducing tetracycline degradation in an alkaline environment. Chlortetracycline showed significantly lower stability compared to tetracycline, which may indicate its greater susceptibility to degradation or epimerization processes. Even though the same procedure was used to prepare the formulations and samples for HPLC analysis, the drug concentrations in the basic and neutral samples were lower than the expected level of about 10 μg/mL on the first day. The slightly acidic formulations (1ChA, 1ChB) showed the highest stability, with a slight decrease in drug concentration to 8.4 μg/mL after 28 days. Formulations with a pH close to neutral (2ChA, 2ChB) showed moderate stability, with chlortetracycline concentrations decreasing from nearly 8 μg/mL on the first day of observation to approximately 5 μg/mL after 28 days. The alkaline formulations (3ChA, 3ChB) showed the greatest susceptibility to degradation. The drug concentration dropped sharply to 1.45 μg/mL for 3ChA and 0.73 μg/mL for 3ChB on 7th day of observation, reaching values close to zero in the following weeks. Azeloglycine did not significantly affect the stability of chlortetracycline in any of the formulations.

The presence of azeloglycine affected the rheological properties of the formulations tested. All formulations exhibited non-Newtonian viscoelastic properties, manifested by a shear-thinning effect in which viscosity decreased with increasing shear rate, in agreement with previous reports on acrylic acid-based polymers [27,28,29]. The addition of azeloglycine reduced the viscosity of all hydrogels tested, regardless of the pH of the environment, indicating its effect on the polymer network structure. Viscosity values for formulations without azeloglycine averaged approximately 215,000 mPas, while those with azeloglycine averaged approximately 115,000 mPas. These changes are not substantial enough to potentially interfere with application of the formulations to the skin surface. The type of antibiotic used had no effect on the viscosity values, confirming that the differences in rheology were dominated by the presence of azeloglycine and the properties of the polymer matrix. The reduced viscosity of hydrogels with azeloglycine could potentially increase their therapeutic efficacy by facilitating skin penetration and interaction with the skin surface, making these formulations particularly promising for dermatological applications.

The effect of azeloglycine on the polymer network structure can also be seen in subtle differences in the FTIR spectra. The broad band in the 3200–3600 cm^−1^ range, associated with O–H stretching vibrations, was less intense in hydrogels containing azeloglycine (Group A) compared to formulations without this component (Group B). This observation suggests that the presence of azeloglycine may reduce the amount of water bound in the polymer network. Analysis of FTIR spectra taken one day after freeze–drying and after 33 days of storage showed no significant differences. This result may indicate high stability of the polymer network and no significant changes in its structure over time.

Another important element of the formulated products is the presence of 2-amino-2-methyl-1,3-propandiol (AMPD), which has activity against free sebum fatty acids. Accumulated sebum inside the hair follicle promotes excessive proliferation of the microbiota, leading to inflammation and painful skin lesions. These deposits limit the effectiveness of therapeutic agents used to eliminate bacteria and reduce inflammation. The sebum model is an important tool in the study of epidermal lipid barrier function and in the development of dermatological and cosmetic products. Its composition must be as close as possible to that of human sebum [85,86,87,88,89,90,91]. Its use allows research to be carried out under controlled laboratory conditions, greatly increasing repeatability and accuracy. Stefaniak et al. analyzed numerous publications on model skin sebum and assessed the extent to which the proposed formulations were compatible with the natural composition of human sebum [88,91]. The model sebum composition proposed by Kubis and Musial and used in the following study was found by Stefaniak to be one of the closest to natural skin sebum [88,92]. The results of previous studies suggest that alcoholamines react with model components of sebum, such as stearic acid, leading to their saponification, and by binding water, the formation of a loose layer of reaction products that can facilitate sebum removal (Equation (1)) [31]:

R1–COOH + R2–NH_2_(aq) → R1–COO– [NH_3_ + –R2](aq) (1)

This process may improve the penetration of the applied active ingredients into the hair follicle and enhance the elimination of pathogens such as *C. acnes* and *S. aureus*, potentially increasing the efficacy of anti-acne therapy. Based on previous studies, it is known that an increase in formulation alkalinity resulting from an increase in AMPD content in hydrogels leads to a greater reactivity of these gels towards model sebum components [31]. This phenomenon may be related to the presence of a greater number of free AMPD molecules not bound to the carboxyl groups of the carbomer. Nevertheless, it was observed that the 3TA and 3ChA gels, despite having the highest pH of approximately 8.2, had slightly lower activity against sebum compared to the 2TA and 2ChA gels, which had a pH of approximately 7.3. After 72 h of observation, samples with the 2TA and 2ChA gels had an average product layer depth of 1.81 mm, while the 3TA and 3ChA gels had an average layer depth of 1.58 mm. A possible explanation for this phenomenon may be the formation of a dense layer of saponification reaction products on the sebum surface, which may inhibit further penetration of the gel into the sebum [23]. Excessive alkaline pH can inhibit the presumed process of saponification of sebum deposits. At the same time, the reduced viscosity of preparations containing azeloglycine compared to analogous gels without this ingredient may translate into increased activity against model sebum. It was observed that the presence of azeloglycine promoted the formation of a deeper layer of reaction products compared to gels without azeloglycine. The product layer for azeloglycine-free gels 2TB, 2ChB, 3TB and 3ChB was significantly lower, averaging 0.83 mm after 72 h. This beneficial effect due to the presence of azeloglycine allows the pH of the formulation to be lowered while maintaining high efficacy against model sebum, making it a valuable ingredient in the development of advanced dermatological formulations. Optimization of the formulation pH is extremely important, as the slightly acidic hydrogels (1TA, 1TB, 1ChA, 1ChB) did not show any reaction within the 72 h study period. For the neutral and mildly alkaline formulations, the highest reaction rate was observed within the first 24 h, which may indicate a gradual depletion of free AMPD in the hydrogels as the reaction progressed. Formulations containing chlortetracycline (2ChA, 3ChA) showed activity similar to that of tetracycline (2TA, 3TA), indicating that the type of antibiotic does not affect the rate of reaction with sebum. Optimization of hydrogel formulations should be performed with respect to pH, with neutral pH formulations containing azeloglycine showing the best reactive properties towards model sebum.

The presence of azeloglycine in the tested formulations also proved to be interesting in the context of the microbiological analyses performed. While *Cutibacterium acnes* is a primary etiological factor in acne vulgaris, its application in in vitro biofilm studies is limited by its anaerobic growth requirements and weak, slow-growing biofilm formation un-der standard laboratory conditions. These features make it difficult to achieve robust, re-producible results, particularly when assessing subtle formulation-dependent effects such as the influence of azeloglycine on antimicrobial performance. As a result, *S. aureus* was selected for the present study. This species is frequently detected in acne lesions as a secondary colonizer and contributes to the inflammatory process through biofilm formation and immune modulation. Moreover, a substantial proportion of *S. aureus* strains remain susceptible to tetracyclines, including tetracycline hydrochloride and chlortetracycline, making it a pharmacologically relevant substitute in this context. Its reliable growth and biofilm-forming capacity under aerobic conditions also enable more standardized, sensitive comparison of hydrogel variants. The outcomes presented in Figure 10 and Figure 12 show a clear distinction in the sensitivity of the models. In the disk diffusion model (Figure 10), no statistically significant differences were observed between hydrogels containing azeloglycine and those without it. This lack of differentiation may be attributed to the physical limitations of the method itself. In this setup, a fixed amount of hydrogel was deposited into an 8 mm well on the surface of solid Mueller–Hinton agar, and diffusion of active compounds occurred in a relatively uniform manner. As the compounds must diffuse through a dense agar matrix, any subtle variations in chemical composition—such as the addition of azeloglycine—may not substantially affect the size of the inhibition zone unless the agent has strong, readily diffusible antimicrobial properties. In contrast, the artificial sebum biofilm model (Figure 12) presents a more physiologically relevant and sensitive environment that may amplify the functional differences between formulations. Here, the hydrogels interact with mature *Staphylococcus aureus* biofilms formed within a lipid-rich matrix. The anti-biofilm effect is evaluated based on metabolic activity, which reflects deeper functional interactions beyond simple surface-level inhibition. In this model, statistically significant differences were observed between hydrogel variants (e.g., 1ChA vs. 1ChB), suggesting that the presence of azeloglycine or other compositional changes influences biofilm eradication capacity, possibly through modulation of biofilm matrix penetration, interference with bacterial adhesion, or disruption of quorum sensing mechanisms, none of which are captured by the standard agar diffusion assay. Together, these findings underscore the importance of using complementary models: the disk diffusion test may be useful for general screening of antimicrobial activity, whereas the sebum biofilm model provides more nuanced insights into how hydrogel composition affects performance in environments that more closely mimic in vivo conditions.

Most importantly, the antibiotic-containing hydrogels showed no toxic effects on *Galleria mellonella* larvae, confirming their biocompatibility. The *G. mellonella* larva model was chosen for local cytotoxicity assessment because it enables biologically meaningful in vivo evaluation of topically applied formulations. In contrast to two-dimensional keratinocyte cultures, which are devoid of extracellular matrix and immune responsiveness, *G. mellonella* larvae possess a protective cuticle and a functional innate immune system. These features allow the detection of complex physiological outcomes such as melanization, behavioral changes, and survival. The absence of toxicity in this model supports the local safety of the tested hydrogels. We regard the Galleria model as a useful intermediate between basic in vitro assays and mammalian studies. In future research, we aim to supplement our findings with results from keratinocyte-based models that offer high translational relevance. This result is crucial, as it indicates that they can be used safely in dermatological therapy, minimizing the risk of adverse skin reactions and allowing their potential use in the treatment of skin diseases. Biocompatibility is the basis for further development of these formulations in clinical practice.

The developed formulations, integrating components with different mechanisms of action, are aimed at obtaining preparations with a complementary therapeutic effect. Their scope of action includes reduction of pathogenic microflora, local anti-inflammatory effect, modulation of pigmentation processes, and improvement in functionality of hair follicle openings. Such multidirectional action can significantly increase the therapeutic efficacy of preparations while minimizing the risk of bacterial resistance and improving the bioactivity of active substances at the site of application.

## 4. Materials and Methods

### 4.1. Reagents

Tetracycline hydrochloride (TC) (Sigma Aldrich, Poznan, Poland), chlortetracycline hydrochloride (ChTC) (Sigma Aldrich), 2-amino-2-methyl-1,3-propanediol AMPD (Sigma Aldrich), Carbopol 980 NF (C 980 NF) polyacrylic acid cross-linked with allyl pentaerythritol (Lubrizol, Wickliffe, OH, USA), potassium azeloyl diglycinate (azeloglycine (A), solution) (Zrob sobie krem, Prochowice, Poland) and demineralized, bi-distilled water were used to prepare the formulations. Lanolin (Fagron, Warsaw, Poland), stearic acid (Sigma Aldrich), cholesterol (Sigma Aldrich), triglycerides of animal origin (Fagron) and squalene (Sigma Aldrich) were used to prepare artificial sebum. Acetonitrile (Sigma Aldrich), formic acid (Sigma Aldrich) and demineralized, bi-distilled water were used in the HPLC analysis. Tetrazolium chloride (Sigma-Aldrich), ethanol and acetic acid (both from POCH, Lublin, Poland) were applied for formazan extraction, while Mueller–Hinton agar powder or broth (Biomaxima, Lublin, Poland) was applied for bacterial cultivation.

### 4.2. Preparation of Hydrophilic Formulations

In this study, 12 hydrogel formulations, each weighing 100 g, were developed. Of these, 6 contained 0.2 g of tetracycline (labeled T) and the remaining 6 contained 0.2 g of chlortetracycline (labeled Ch). In each group, half of the formulations were enriched with 2.0 g azeloglycine (labeled A), while the other half did not contain this substance (labeled B). In each of the two drug groups (T and Ch), two hydrogels were formulated with a pH of approximately 6.60 (labeled 1A and 1B), two with a pH close to 7.30 (labeled 2A and 2B) and two with a pH of approximately 8.20 (labeled 3A and 3B). Control preparations without antibiotics, labeled 0, were developed to assess microbiological activity and for FTIR analysis. The pH values were obtained by varying the AMPD content. The detailed composition of each formulation is shown in Table 2.

To obtain 100 g of hydrogel, weighed amounts of AMPD, Carbopol and water were mixed and conditioned for 24 h at 4 °C. Then, 0.2 g of the appropriate antibiotic, previously dissolved in the specified amount of water, was added to the resulting hydrogel. For preparations marked A, azeloglycine was also added to the formulation. All formulations were homogenized for 20 min using an Eprus U500 automatic pharmacy mixer (Eprus, Bielsko-Biała, Poland) at the lowest speed of 630 rpm. The hydrogels were stored in non-transparent containers at 4 °C.

### 4.3. Measurement of pH Value

The pH values of the formulated preparations were measured using a CPC-505 pH meter (accuracy ± 0.002 pH, Elmetron Sp.j., Zabrze, Poland) and an ERH-11S electrode (Elmetron Sp.j., Zabrze, Poland) designed for viscous preparations such as ointments, creams and gels. Each measurement was repeated five times.

### 4.4. Antibiotic Stability Analysis by HPLC

#### 4.4.1. Preparation of Hydrogel Samples

Chromatographic stability analysis of the antibiotics contained in the formulations was carried out over a 28-day period. HPLC measurements were performed at regular intervals every 7 days. For sample preparation, 0.5 g of gel was taken each time and dissolved in 99.5 g of distilled water, stirring on an Arex Digital Pro magnetic stirrer (Velp Scientifica, Usmate (MB), Italy) for 20 min at 900 rpm. Six 1 mL samples were then taken and subjected to chromatographic analysis.

#### 4.4.2. HPLC Analysis

Analysis of both antibiotics was performed on a Thermo Scientific Dionex UltiMate 3000 instrument (Thermo Scientific Dionex, Sunnyvale, CA, USA), which included a DAD-3000 UV detector, TCC-3000SD column oven, LPG-3400SD pump module and WPS-3000TSL autosampler. Chromatographic separation was performed using an RP-18 LiChroCART column, 125 mm × 4 mm, 5 μm (Merck, Darmstadt, Germany) at 40 °C. Chromeleon v 7.2 SR5 software (Thermo Scientific Dionex, Sunnyvale, CA, USA) was used for data processing. Both tetracycline and chlortetracycline were determined using the same method. The mobile phase was 0.1% formic acid in water (A) and 0.1% formic acid in acetonitrile (B). The flow rate was 1.0 mL/min and the gradient was as follows: started at 7% B until 0.5 min, then increased up to 50% at 4 min and then to 95% at 5 min. This flow rate was maintained until the 6th minute and then reduced to 7% B at the 7th minute. This flow rate was maintained until 9 min. The retention time for tetracycline was 4.14 min and detection was performed at 280 nm. A series of aqueous solutions of commercial TC were prepared and linearity was observed from 1.1107 μg/mL to 17.3582 μg/mL with a correlation coefficient (R) of 0.9978 (y = 0.3024x – 0.1455). The retention time for chlortetracycline was 4.71 min. The detection was carried out at 280 nm. Linearity was observed at concentrations from 1.0301 μg/mL to 16.5930 μg/mL based on a range of aqueous solutions of commercial chlortetracycline with a correlation coefficient (R) of 0.9993 (y = 0.2160x – 0.0335).

### 4.5. Determination of the Viscosity Curve of the Preparations

Viscosity curves for all formulations were determined using a Brookfield DV-III+ rotational rheometer (AMETEK Brookfield, Middleboro, MA, USA) in a cone/plate system with a CP51 cone. The data obtained were analyzed using Rheocalc v3.2 for Windows software (AMETEK Brookfield, Middleboro, MA, USA). The measurement system included a thermostat to maintain a constant, controlled temperature. Viscosity measurements were performed at 37 °C. Each measurement was taken over a decade of shear rates with a pre-shear period and a stabilized reading before data collection. The shear rate increased during the measurements from 0.38 s^−1^ to 3.84 s^−1^ and the speed increased from 0.1 rpm to 1.0 rpm. The corresponding forward and reverse rheograms were recorded. Each viscosity measurement was repeated five times.

### 4.6. Artificial Skin Sebum Preparation

This study used the model sebum composition developed by Kubis and Musial [24,92]. The detailed formulation with the corresponding natural equivalents is summarized in Table 3.

All the ingredients were weighed out, melted in a water bath, mixed, and allowed to solidify at 23 °C.

### 4.7. Evaluation of Hydrogels’ Activity Against Artificial Sebum Components

Scaled PVC tubes of 4 mm internal diameter were used to assess the activity of the hydrogels in interacting with the components of the model skin sebum. Tubes were filled with 0.5 mL of melted skin sebum and allowed to solidify. This system was designed to simulate a model of a sebum-filled hair follicle. Once the sebum had solidified, 0.5 mL of each hydrogel formulation was applied to the surface and the tube openings were sealed with a plastic compound to prevent the gels from drying out. The thickness of the resulting layer of reaction products was assessed every 24 h for a period of 72 h. The layer thickness was measured using a Powerfix IAN Z22855 digital caliper (accuracy ± 0.02 mm, Milomex Ltd., Bedfordshire, UK). Visual documentation of the system was achieved using a Samsung A53 digital camera (Macro 5 Mpx camera, 1.12 μm, FF, f/2.4). For each hydrogel, 8 measurements were taken.

### 4.8. ATR–FTIR Measurements of Freeze-Dried Formulations

Prior to analysis by attenuated total reflectance–Fourier transform infrared spectroscopy (ATR–FTIR), 30 g of each preparation was frozen and then freeze-dried for 26 h using an Alpha 1-2 LD freeze dryer (Martin Christ Freeze Dryers, Osterode am Harz, Germany). FTIR spectra were recorded using a spectrometer with an ATR attachment (Nicolet iS50, Thermo Scientific, Waltham, MA, USA). The resulting data were analyzed using OMNIC software (v 9, Thermo Fisher Scientific, Waltham, MA, USA). The FTIR spectra were recorded with 32 scans per sample cycle. The wave number resolution was 4 cm^−1^ in the wavelength range from 4000 to 400^−1^. ATR–FTIR spectra of the lyophilized formulations and the substrates in commercial form were measured at ambient temperature. All measurements were taken under the same conditions 1 day after freeze-drying and 33 days after freeze-drying. Between measurements, the lyophilized hydrogels were stored at 4 °C in opaque containers.

### 4.9. Assessment of Staphylococcal Growth Inhibition Zone After Exposure on Hydrogels Using Modified Disk Diffusion Method

*Staphylococcus aureus* strain ATCC 6538 was cultivated in liquid tryptic soy broth (TSB, Biomaxima, Lublin, Poland) medium at 37 °C/24 h. Next, microbial suspension was diluted using densitometry (Den-1, Biosan, Piła, Poland) to a density of 0.5 McFarland and spread on the Mueller–Hinton (MH, Biomaxima, Lublin, Poland) agar plate, as is performed for antibiotic sensitivity assessment in the disk diffusion method. After spreading, a hole of 8 mm diameter was cut in the center of the plate using a JLB 320 cork-borer device (Boehm, La Fouillouse, France). Next, the MH plate was placed in the weight device (AS 110 R2 PLUS, Radwag, Radom, Poland). Hydrogel (500 ± 10 mg) was then introduced to the 8 mm hole in the MH plate. The whole set was then incubated at 37 °C/24 h. After incubation, plates were photographed in a photographic chamber (Puluz Photo Light Box, Shenzhen, China). The captured photos were uploaded and processed in ImageJ software (version 1.54p, NIH Bethesda, MD, USA) to determine the area (in mm^2^) of the inhibition zone. The analysis was performed in the following steps: the photo was opened in the program -> a 10 mm segment was precisely marked on the ruler, and then the following commands were performed: analyze–set scale–known distance 10 mm–freehand (of inhibition zone)–analyze–measure.

### 4.10. Assessment of Staphylococcal Biofilm Reduction in the Artificial Sebum

Artificial sebum (4 mL) was placed in the wells of a 6-well plate and left to solidify. Next, 2 mL of 10^5^ staphylococcal CFU-containing M-H solution was introduced and incubated at 37 °C/24 h. After 24 h, the medium was replaced by a fresh one and cell strainer inserts (Sigma-Aldrich, Darmstadt, Germany) containing 500 mg of each hydrogel were placed on the top of the well. Next, the whole setting was incubated at 37 °C/24 h. After incubation, the inserts were removed, and the medium was replaced with the fresh one containing tetrazolium chloride (TTC, Sigma-Aldrich, Darmstadt, Germany) at 1% concentration. Next, the plates were incubated for 2 h at 37 °C. During these 2 h, live biofilm-forming *S. aureus* cells metabolized colorless TTC into red formazan. The formazan was extracted using a solution consisting of ethanol and acetic acid in a ratio of 90:10 (*v*/*v*), respectively (POCH, Lublin, Poland), and the level of absorbance of the formazan was measured at a wavelength of 490 nm. The control of this experiment was the setting in which no antimicrobial was added. The results of the absorbance for such a control setting were considered to be 100% and were used to calculate the biofilm eradication by means of the following formula: biofilm eradication (%) = 100% x (value of the hydrogel-exposed sample absorbance/value of nonexposed sample absorbance).

### 4.11. Assessment of Hydrogels’ Local Cytotoxicity Towards Galleria mellonella Larvae

Larvae of the greater wax moth, *G. mellonella*, of average weight equal to 0.20 ± 0.2 g, were selected for the experiment. The larvae were immobilized as described in Patent application: P.451281: *Stabilization module, multi-plane tripod, and modular system for immobilizing and nourishing larvae under controlled incubation conditions.* Approximately 100 mg of tested hydrogels was placed on the dorsal site of larvae. Cellulosic hydrogel soaked with phosphate-buffered saline or 70% ethanol served as control of survival or the method’s usability control, respectively. The larvae were placed in 90 mm petri dishes and incubated at 30 °C/5 days. Each day, the mortality of the larvae was monitored. Death was defined when the larvae were nonmobile, melanized, and did not react to physical stimuli.

### 4.12. Statistical Analysis

Statistical analyses were performed using GraphPad Prism 10 (San Diego, CA, USA). The normality of distribution was verified using the Shapiro–Wilk test. Analysis of variance (ANOVA) was performed to assess statistical significance. For multiple comparisons, Tukey’s post hoc test was applied. A *p*-value threshold of less than 0.05 was set for significance in the ANOVA. For the Tukey post hoc analysis, significance levels were further categorized as *p* < 0.001 for specific pairwise comparisons.

## 5. Conclusions

This research indicates the potential of hydrogel formulations containing tetracycline and azeloglycine as modern and advanced anti-acne preparations. Tetracycline has been shown to have higher stability compared to chlortetracycline, especially in mildly acidic and neutral environments, highlighting its greater utility in the development of topical dermatological products. The addition of azeloglycine improved the rheological properties of the hydrogels and reduced tetracycline degradation in an alkaline environment, while promoting better skin penetration and interaction with the skin structure. Microbiological analyses showed high antimicrobial efficacy of the developed formulations against *S. aureus* strains, confirming their ability to inhibit bacterial growth. In addition, biocompatibility tests on *G. mellonella* larvae showed no local toxicity, demonstrating the safety of the formulations and their potential use in dermatological therapy. These formulations may have potential complementary effects, combining antimicrobial, anti-inflammatory, and hair follicle cleansing support. Due to these properties, the developed formulations represent an innovative solution for the topical treatment of acne, offering a comprehensive therapeutic approach. The introduction of azeloglycine provides a favorable physicochemical and rheological profile, making the hydrogels not only effective but also user-friendly and promising for clinical applications. In future studies, we plan to further analyze the effect of additives such as azeloglycine on the molecular structure of polymers to optimize their rheological properties and adjust them to specific therapeutic requirements. A crucial step will also be to conduct detailed clinical trials to evaluate the efficacy and safety of these formulations in the practical treatment of patients with acne. The results highlight the potential of hydrogel formulations in the treatment of skin diseases associated with excessive sebum secretion, such as acne, opening up new perspectives in topical dermatology.

## Figures and Tables

**Table 1:** The pH values of all hydrogels, *p* < 0.0001. T, tetracycline; Ch, chlortetracycline; A, presence of azeloglycine; B, absence of azeloglycine.

Formulation	pH Value ± SD
1TA	6.41 ± 0.01
1TB	6.63 ± 0.05
1ChA	6.62 ± 0.02
1ChB	6.72 ± 0.01
2TA	7.20 ± 0.01
2TB	7.45 ± 0.01
2ChA	7.21 ± 0.01
2ChB	7.35 ± 0.01
3TA	8.19 ± 0.01
3TB	8.29 ± 0.01
3ChA	8.17 ± 0.02
3ChB	8.24 ± 0.02

**Table 2:** Composition of all hydrogel formulations. TC—tetracycline hydrochloride, ChTC—chlortetracycline hydrochloride, AMPD—2-amino-2-methyl-1,3-propanediol. T—tetracycline, Ch—chlortetracycline, A—presence of azeloglycine, B—absence of azeloglycine.

Formulation	TC [g]	ChTC [g]	Azeloglycine [g]	AMPD [g]	Carbopol 980 NF [g]	Water [g]
1TA	0.2	0.0	2.0	0.7	0.7	96.4
2TA	0.2	0.0	2.0	0.9	0.7	96.2
3TA	0.2	0.0	2.0	1.1	0.7	96.0
1TB	0.2	0.0	0.0	0.7	0.7	98.4
2TB	0.2	0.0	0.0	0.9	0.7	98.2
3TB	0.2	0.0	0.0	1.1	0.7	98.0
1ChA	0.0	0.2	2.0	0.7	0.7	96.4
2ChA	0.0	0.2	2.0	0.9	0.7	96.2
3ChA	0.0	0.2	2.0	1.1	0.7	96.0
1ChB	0.0	0.2	0.0	0.7	0.7	98.4
2ChB	0.0	0.2	0.0	0.9	0.7	98.2
3ChB	0.0	0.2	0.0	1.1	0.7	98.0
1.0A	0.0	0.0	2.0	0.7	0.7	96.6
2.0A	0.0	0.0	2.0	0.9	0.7	96.4
3.0A	0.0	0.0	2.0	1.1	0.7	96.2
1.0B	0.0	0.0	0.0	0.7	0.7	98.6
2.0B	0.0	0.0	0.0	0.9	0.7	98.4
3.0B	0.0	0.0	0.0	1.1	0.7	98.2

**Table 3:** Components of model skin sebum.

Components	Artificial Skin Sebum
Cholesterol	4%
Squalene	12%
Stearic acid as a free fatty acid	24%
Lanolin as wax	26%
Pork lard as triglyceride	34%

## Data Availability

Data are contained within the article.

## Abbreviations

The following abbreviations are used in this manuscript:

AMPD	2-amino-2-methyl-1,3-propandiol
ChTC	chlortetracycline hydrochloride
ChTA	formulation with chlortetracycline and azeloglycine
ChTB	formulation with chlortetracycline and without azeloglycine
FTIR	Fourier transform infrared spectroscopy
HPLC	high-performance liquid chromatography
TC	tetracycline hydrochloride
TA	formulation with tetracycline and azeloglycine
TB	formulation with tetracycline and without azeloglycine

## References

[1] Dréno B. (2010). Recent Data on Epidemiology of Acne. Ann. Dermatol. Venereol..

[2] Fried R.G., Wechsler A. (2006). Psychological Problems in the Acne Patient. Dermatol. Ther..

[3] Reynolds R.V., Yeung H., Cheng C.E., Cook-Bolden F., Desai S.R., Druby K.M., Freeman E.E., Keri J.E., Stein Gold L.F., Tan J.K.L. (2024). Guidelines of Care for the Management of Acne Vulgaris. J. Am. Acad. Dermatol..

[4] Dréno B., Dagnelie M.A., Khammari A., Corvec S. (2020). The Skin Microbiome: A New Actor in Inflammatory Acne. Am. J. Clin. Dermatol..

[5] Lee Y.B., Byun E.J., Kim H.S. (2019). Potential Role of the Microbiome in Acne: A Comprehensive Review. J. Clin. Med..

[6] Farrar M.D., Ingham E. (2004). Acne: Inflammation. Clin. Dermatol..

[7] Schwartz Robert A. (2010). Al-Mutairi Nawaf Topical Antibiotics in Dermatology An Update. Gulf J. Dermatol. Venereol..

[8] Drake L., Reyes-Hadsall S., Barbieri J.S., Mostaghimi A. (2022). New Developments in Topical Acne Therapy. Am. J. Clin. Dermatol..

[9] Katsambas A., Papakonstantinou A. (2004). Acne: Systemic Treatment. Clin. Dermatol..

[10] Zaenglein A.L., Pathy A.L., Schlosser B.J., Alikhan A., Baldwin H.E., Berson D.S., Bowe W.P., Graber E.M., Harper J.C., Kang S. (2016). Guidelines of Care for the Management of Acne Vulgaris. J. Am. Acad. Dermatol..

[11] Tsankov N., Broshtilova V., Kazandjieva J. (2004). Tetracyclines in Dermatology. Disease-a-Month.

[12] Kim Y.S., Kim H.S. (2024). Tetracyclines Revisited: Tetracyclines in the Field of Dermatology. Dermatology.

[13] Dawson C.R., Daghfous T., Messadi M., Hoshiwara I., Vastine D.W., Yoneda C., Schacter J. (1974). Severe Endemic Trachoma in Tunisia II. A Controlled Therapy Trial of Topically Applied Chlortetracycline and Erythromycin. Arch. Ophthalmol..

[14] Burton J. (1990). A Placebo-Controlled Study to Evaluate the Efficacy of Topical Tetracycline and Oral Tetracycline in the Treatment of Mild to Moderate Acne. J. Int. Med. Res..

[15] Navarro-Triviño F.J., Pérez-López I., Ruíz-Villaverde R. (2020). Doxycycline, an Antibiotic or an Anti-Inflammatory Agent? The Most Common Uses in Dermatology. Actas Dermosifiliogr..

[16] Toyoda M., Morohashi M. (1998). An Overview of Topical Antibiotics for Acne Treatment. Dermatology.

[17] Sapadin A.N., Fleischmajer R. (2006). Tetracyclines: Nonantibiotic Properties and Their Clinical Implications. J. Am. Acad. Dermatol..

[18] Sieber M.A., Hegel J.K.E. (2013). Azelaic Acid: Properties and Mode of Action. Skin. Pharmacol. Physiol..

[19] Webster G. (2000). Combination Azelaic Acid Therapy for Acne Vulgaris. J. Am. Acad. Dermatol..

[20] Sadeghzadeh-Bazargan A., Behrangi E., Najar Nobari N., Ghassemi M., Roohaninasab M., Goodarzi A. (2022). Systematic Review of Clinical Studies Assessing the Needling for Treatment of Melasma: Focusing on Efficacy, Safety, and Recurrence Rate. J. Cosmet. Dermatol..

[21] Maramaldi G., Esposito M.A. (2008). Potassium Azeloyl Diglycinate: A Multifunctional Skin Lightener. Cosmet. Toilet..

[22] Rohmani S., Dinda K.E., Ainurofiq A. (2021). Formulation and Evaluation of the Cream Made from Potassium Azeloyl Diglycinate as an Anti-Aging. J. Phys. Conf. Ser..

[23] Kostrzębska A., Musiał W. (2020). The Influence of Increasing Concentrations of AMPD on the Efficacy of Its Penetration into a Model Skin Sebum Layer. Pharmaceutics.

[24] Musial W., Kubis A. (2006). Preliminary Evaluation of Interactions between Selected Alcoholamines and Model Skin Sebum Components. Chem. Pharm. Bull..

[25] Kostrzębska A., Złocińska A., Musiał W. (2021). Evaluation of the Influence of a Hydrogel Containing AMPD on the Stability of Tetracycline Hydrochloride. Pharmaceutics.

[26] Wróblewska M., Słyż J., Winnicka K. (2019). Rheological and Textural Properties of Hydrogels, Containing Sulfur as a Model Drug, Made Using Different Polymers Types. Polim. Polym..

[27] Varges P.R., Costa C.M., Fonseca B.S., Naccache M.F., De Souza Mendes P.R. (2019). Rheological Characterization of Carbopol ® Dispersions in Water and in Water/Glycerol Solutions. Fluids.

[28] Kim J.Y., Song J.Y., Lee E.J., Park S.K. (2003). Rheological Properties and Microstructures of Carbopol Gel Network System. Colloid Polym. Sci..

[29] Shafiei M., Balhoff M., Hayman N.W. (2018). Chemical and Microstructural Controls on Viscoplasticity in Carbopol Hydrogel. Polymer.

[30] Curran S.J., Hayes R.E., Afacan A., Williams M.C., Tanguy P.A. (2002). Properties of Carbopol Solutions as Models for Yield-Stress Fluids. J. Food Sci. Food Eng. Phys. Prop..

[31] Kostrzębska A., Pączek K., Weselak A., Musiał W. (2023). Effect of Hydrogel Substrate Components on the Stability of Tetracycline Hydrochloride and Swelling Activity against Model Skin Sebum. Int. J. Mol. Sci..

[32] Marchetti M., Perini A., Zanella M., Benetti F., Donelli D. (2024). Study on the Fate of the Carbopol® Polymer in the Use of Hand Sanitizer Gels: An Experimental Model to Monitor Its Physical State from Product Manufacturing up to the Final Hand Rinse. Microplastics.

[33] Kirwan L.J., Fawell P.D., Van Bronswijk W. (2003). In Situ FTIR-ATR Examination of Poly(Acrylic Acid) Adsorbed onto Hematite at Low PH. Langmuir.

[34] Islam M.T., Rodríguez-Hornedo N., Ciotti S., Ackermann C. (2004). Fourier Transform Infrared Spectroscopy for the Analysis of Neutralizer-Carbomer and Surfactant-Carbomer Interactions in Aqueous, Hydroalcoholic, and Anhydrous Gel Formulations. Am. Assoc. Pharm. Sci. J..

[35] Sahoo S., Chakraborti C.K., Mishra S.C., Nanda U.N. (2011). Qualitative Analysis of Environmentally Responsive Biodegradable Smart Carbopol Polymer. Int. J. Pharm. Sci. Rev. Res..

[36] Sahoo S., Chakraborti C.K., Mishra S.C., Naik S., Nanda U.N. (2011). FTIR and Raman Spectroscopy as a Tool for Analyzing Sustained Release Hydrogel of Ciprofloxacin/Carbopol Polymer. IJPSR.

[37] Taleb M.A., Hussien A.M., Al-Fiky A.F., El-Sayed H. (2022). Preparation of Fatty Acid-Amino Diol Condensate and Its Utilization as a Durable Nonionic Softener for PAN Fabrics. Heliyon.

[38] Kobryń J., Demski P., Raszewski B., Zięba T., Musiał W. (2024). Effect of Co-Solvents, Modified Starch and Physical Parameters on the Solubility and Release Rate of Cryptotanshinone from Alcohologels. Molecules.

[39] Hou H., Dai Z., Liu X., Yao Y., Liao Q., Yu C., Li D. (2018). Reutilization of the Expired Tetracycline for Lithium Ion Battery Anode. Sci. Total Environ..

[40] Mikulski C.M., Fleming J., Fleming D., Karayannis N.M. (1988). Chelates of Tetracycline with First Row Transition Metal Perchlorates. Inorganica Chim. Acta..

[41] Apiwongngam J., Limwikrant W., Jintapattanakit A., Jaturanpinyo M. (2018). Enhanced Supersaturation of Chlortetracycline Hydrochloride by Amorphous Solid Dispersion. J. Drug Deliv. Sci. Technol..

[42] Chen G., Zhao L., Dong Y. (2011). hua Oxidative Degradation Kinetics and Products of Chlortetracycline by Manganese Dioxide. J. Hazard. Mater..

[43] Caminati G., Focardi C., Gabrielli G., Gambinossi F., Mecheri B., Nocentini M., Puggelli M. (2002). Spectroscopic Investigation of Tetracycline Interaction with Phospholipid Langmuir-Blodgett Films. Mater. Sci. Eng. C.

[44] Li Z., Kolb V.M.K., Jiang W.T., Hong H. (2010). FTIR and XRD Investigations of Tetracycline Intercalation in Smectites. Clays Clay Min..

[45] Chang P.H., Jean J.S., Jiang W.T., Li Z. (2009). Mechanism of Tetracycline Sorption on Rectorite. Colloids Surf. A Physicochem. Eng. Asp..

[46] Nandiyanto A.B.D., Ragadhita R., Fiandini M. (2023). Interpretation of Fourier Transform Infrared Spectra (FTIR): A Practical Approach in the Polymer/Plastic Thermal Decomposition. Indones. J. Sci. Technol..

[47] Nattkemper A., Schleiden T., Migliavacca J.M., Melin T. (2003). Monitoring Crystallization Kinetics of Azelaic Acid by in Situ FTIR Spectroscopy in Three-Phase Systems. Chem. Eng. Technol..

[48] Kumar S., Rai A.K., Singh V.B., Rai S.B. (2005). Vibrational Spectrum of Glycine Molecule. Spectrochim. Acta A Mol. Biomol. Spectrosc..

[49] Santoro M., Marchetti P., Rossi F., Perale G., Castiglione F., Mele A., Masi M. (2011). Smart Approach to Evaluate Drug Diffusivity in Injectable Agar-Carbomer Hydrogels for Drug Delivery. J. Phys. Chem. B.

[50] Tan J.K.L., Bhate K. (2015). A Global Perspective on the Epidemiology of Acne. Br. J. Dermatol..

[51] Juhl C.R., Bergholdt H.K.M., Miller I.M., Jemec G.B.E., Kanters J.K., Ellervik C. (2018). Dairy Intake and Acne Vulgaris: A Systematic Review and Meta-Analysis of 78,529 Children, Adolescents, and Young Adults. Nutrients.

[52] Zagórska-Dziok M., Sobczak M. (2020). Hydrogel-Based Active Substance Release Systems for Cosmetology and Dermatology Application: A Review. Pharmaceutics.

[53] Teng Y., Li S., Tang H., Tao X., Fan Y., Huang Y. (2023). Medical Applications of Hydrogels in Skin Infections: A Review. Infect. Drug Resist..

[54] Pavlačková J., Pecháčková H., Egner P., Mokrejš P., Gál R., Janalíková M. (2023). The Effect of Cosmetic Treatment and Gel Laser Therapy on the Improvement of Comedogenic Skin Type. Gels.

[55] Chopra I., Roberts M. (2001). Tetracycline Antibiotics: Mode of Action, Applications, Molecular Biology, and Epidemiology of Bacterial Resistance. Microbiol. Mol. Biol. Rev..

[56] Roberts M.C. (2003). Tetracycline Therapy: Update. Clin. Infect. Dis..

[57] Solomons B. (1951). Aureomycin: Its Topical Use In Some Skin Diseases. Br. Med. J..

[58] Darougar S., Viswalingam N., El-Sheikh H., Hunter P.A., Yearsley P. (1981). A Double-Blind Comparison of Topical Therapy of Chlamydial Ocular Infection (TRIC Infection) with Rifampicin or Chlortetracycline. Br. J. Ophthalmol..

[59] Esposito S., Bassetti M., Concia E., De Simone G., De Rosa F.G., Grossi P., Novelli A., Menichetti F., Petrosillo N., Tinelli M. (2017). Diagnosis and Management of Skin and Soft-Tissue Infections (SSTI). A Literature Review and Consensus Statement: An Update. J. Chemother..

[60] Karadag A.S., Aslan Kayıran M., Wu C.Y., Chen W., Parish L.C. (2021). Antibiotic Resistance in Acne: Changes, Consequences and Concerns. J. Eur. Acad. Dermatol. Venereol..

[61] Fernandez-Obregon A.C., Rohrback J., Reichel M.A., Willis C. (2005). Current Use of Anti-Infectives in Dermatology. Expert Rev. Anti. Infect. Ther..

[62] Ochsendorf F. (2010). Minocycline in Acne Vulgaris Benefits and Risks. Am. J. Clin. Dermatol..

[63] Le C.-Y., Ye Y.-J., Xu J., Li L., Feng X.-Q., Chen N.-P., Zhu B.-Q., Ding Z.-S., Qian C.-D. (2023). Hinokitiol Selectively Enhances the Antibacterial Activity of Tetracyclines against Staphylococcus Aureus. Microbiol. Spectr..

[64] Luhaibi D.K., Ali H.H.M., Al-Ani I., Shalan N., Al-Akayleh F., Al-Remawi M., Nasereddin J., Qinna N.A., Al-Adham I., Khanfar M. (2023). The Formulation and Evaluation of Deep Eutectic Vehicles for the Topical Delivery of Azelaic Acid for Acne Treatment. Molecules.

[65] Berardesca E., Iorizzo M., Abril E., Guglielmini G., Caserini M., Palmieri R., Piérard G.E. (2012). Clinical and Instrumental Assessment of the Effects of a New Product Based on Hydroxypropyl Chitosan and Potassium Azeloyl Diglycinate in the Management of Rosacea. J. Cosmet. Dermatol..

[66] Kostrzębska A., Szczepaniak G. (2024). Anti-Acne Preparations Containing Tetracycline, Azelaic Acid and Azeloglycine: Optimization of Stability and Physicochemical Properties. Polim. Med..

[67] Wu Y., Fassihi R. (2005). Stability of Metronidazole, Tetracycline HCl and Famotidine Alone and in Combination. Int. J. Pharm..

[68] Pena A., Carmona A., Barbosa A., Lino C.M., Silveira M.I., Castillo B. (1998). Determination of Tetracycline and Its Major Degradation Products by Liquid Chromatography with Fluorescence Detection. J. Pharm. Biomed. Anal..

[69] Cervini P., MacHado L.C.M., Ferreira A.P.G., Ambrozini B., Cavalheiro É.T.G. (2016). Thermal Decomposition of Tetracycline and Chlortetracycline. J. Anal. Appl. Pyrolysis.

[70] Gajda A., Posyniak A. (2009). Tetracyclines and Their Epimers in Animal Tissues by High-Performance Liquid Chromatography. Bull. Vet. Inst. Pulawy.

[71] Jia A., Xiao Y., Hu J., Asami M., Kunikane S. (2009). Simultaneous Determination of Tetracyclines and Their Degradation Products in Environmental Waters by Liquid Chromatography-Electrospray Tandem Mass Spectrometry. J. Chromatogr. A.

[72] Halling-Sørensen B., Sengeløv G., Tjørnelund J. (2002). Toxicity of Tetracyclines and Tetracycline Degradation Products to Environmentally Relevant Bacteria, Including Selected Tetracycline-Resistant Bacteria. Arch. Environ. Contam. Toxicol..

[73] Keßler D.N., Fokuhl V.K., Petri M.S., Spielmeyer A. (2019). Abiotic Transformation Products of Tetracycline and Chlortetracycline in Salt Solutions and Manure. Chemosphere.

[74] Pena A., Palilis L.P., Lino C.M., Silveira M.I., Calokerinos A.C. (2000). Determination of Tetracycline and Its Major Degradation Products by Chemiluminescence. Anal. Chim. Acta..

[75] Hoener B.-A., Sokoloski T.D., Mitscher L.A., Malspeis L. (1974). Kinetics of Dehydration of Epitetracycline in Solution. J. Pharm. Sci..

[76] Kelly R.G. (1964). Determination of Anhydrotetracycline and 4-Epianhydrotetracycline in a Tetracycline Mixture. J. Pharm. Sci..

[77] Hasan T., Allen M., Cooperman B.S. (1985). Anhydrotetracycline Is a Major Product of Tetracycline Photolysis. J. Org. Chem..

[78] Davies A.K., McKellar J.F., Phillips G.O., Reid A.G. (1979). Photochemical Oxidation of Tetracycline in Aqueous Solution. J. Chem. Soc. Perkin Trans..

[79] Oka H., Ikai Y., Kawamura N., Yamada M., Harada K., Ito S., Suzuki M. (1989). Photodecomposition Products of Tetracycline in Aqueous Solution. J. Agric. Food Chem..

[80] Kennedy D.G., McCracken R.J., Carey M.P., Blanchflower W.J., Hewitt S.A. (1998). Iso- and Epi-Iso-Chlortetracycline Are the Principal Metabolites of Chlortetracycline in the Hen’s Egg. J. Chromatogr. A.

[81] Gómez-Pacheco C.V., Sánchez-Polo M., Rivera-Utrilla J., López-Peñalver J.J. (2012). Tetracycline Degradation in Aqueous Phase by Ultraviolet Radiation. Chem. Eng. J..

[82] López-Peñalver J.J., Sánchez-Polo M., Gómez-Pacheco C.V., Rivera-Utrilla J. (2010). Photodegradation of Tetracyclines in Aqueous Solution by Using UV and UV/H_2_O_2_ Oxidation Processes. J. Chem. Technol. Biotechnol..

[83] Drexel R.E., Olack G., Jones C., Santim R., Morrison H., Chmurny G. (1990). Lumitetracycline: A Novel New Tetracycline Photoproduct. J. Org. Chem..

[84] Hasan T., Kochevar I.E., McAuliffe D.J., Cooperman B.S., Abdulah D. (1984). Mechanism of Tetracycline Phototoxicity. J. Investig. Dermatol..

[85] Pappas A. (2009). Epidermal Surface Lipids. Derm. Endocrinol..

[86] Gerhardt L.C., Schiller A., Müller B., Spencer N.D., Derler S. (2009). Fabrication, Characterisation and Tribological Investigation of Artificial Skin Surface Lipid Films. Tribol. Lett..

[87] Smith K.R., Thiboutot D.M. (2008). Skin Lipids. Sebaceous Gland Lipids: Friend or Foe?. J. Lipid. Res..

[88] Stefaniak A.B., Harvey C.J. (2006). Dissolution of Materials in Artificial Skin Surface Film Liquids. Toxicol. Vitr..

[89] Jia Y., Gan Y., He C., Chen Z., Zhou C. (2018). The Mechanism of Skin Lipids Influencing Skin Status. J. Dermatol. Sci..

[90] Knox S., O’Boyle N.M. (2021). Skin Lipids in Health and Disease: A Review. Chem. Phys. Lipids.

[91] Stefaniak A.B., Harvey C.J., Wertz P.W. (2010). Formulation and Stability of a Novel Artificial Sebum under Conditions of Storage and Use. Int. J. Cosmet. Sci..

[92] Musial W., Kubis A. (2003). Preliminary Assessment of Alginic Acid as a Factor Buffering Triethanolamine Interacting with Artificial Skin Sebum. Eur. J. Pharm. Biopharm..

